# The Clinical Efficacy Analysis of Treatment With a Willis Covered Stent in Traumatic Pseudoaneurysm of the Internal Carotid Artery

**DOI:** 10.3389/fneur.2021.739222

**Published:** 2021-10-06

**Authors:** Yueyuan Zhao, Zhiwen Liu, Ronghui Sun, Li Pan, Ming Yang, Jian Song, Lianting Ma

**Affiliations:** ^1^The First School of Clinical Medicine, Southern Medical University, Guangzhou, China; ^2^Department of Neurosurgery, General Hospital of Central Theater Command, Wuhan, China

**Keywords:** pseudoaneurysm carotid artery, endoleak, Willis covered stent, complications, digital subtraction angiography

## Abstract

**Objective:** To investigate the safety and efficacy of Willis covered stents (WCS) in the treatment of traumatic pseudoaneurysm of the cranial internal carotid artery (CICA).

**Methods:** Fifteen patients with traumatic pseudoaneurysm of the intracranial segment of the ICA treated with the WCS system at our institution from 2013 to 2019 were analyzed retrospectively. Follow-up observation and digital subtraction angiography (DSA) examination were conducted ~6 months after the treatment.

**Results:** DSA performed immediately after stent deployment revealed that complete occlusion of the lesion was achieved in 13 patients and that endoleak occurred in two patients. In 12 patients, postoperative DSA examination indicated that the lesions were completely occluded. In two patients who had a second stent implantation at the break of the ICA, traumatic ICA rupture was essentially completely obstructed in 1 patient. The endoleak remained in one patient with carotid cavernous sinus fistula because the placement of the second stent system was difficult with his ICA tortuosity. No recurrence of aneurysms, hemorrhage, or other lesions was observed, and the patients' parent arteries were patent without stenosis. No procedure-related complications or ischemic strokes occurred during the follow-up period of ~6 months.

**Conclusions:** For treatment of traumatic pseudoaneurysm of the CICA, Willis covered stent implantation in some appropriate cases, is safe and effective. However, large-sample controlled studies and multicenter studies are needed for further confirmation.

## Introduction

Carotid pseudoaneurysm refers to the formation of a hematoma by arterial wall damage and blood extravasation due to various causes. The hematoma gradually dissolves with time. Under the impact of continuous arterial pulsatile pressure, the hematoma communicates with the arterial lumen through the arterial crevasse, forming a pseudoaneurysm. Traumatic pseudoaneurysm of the intracranial segment of the internal carotid artery is the most common type, with an incidence of ~1% of all intracranial aneurysms (1), and the clinical manifestations are recurrent massive nasal bleeding, intracranial hemorrhage, and ischemic stroke. Conventional surgical repair is difficult due to poor intracranial collateral circulation when ligating or occluding the parent artery, which can lead to disability or death of the patient. The Willis covered stent (WCS) is specifically used for the treatment of intracranial cerebrovascular diseases. It consists of two parts: a balloon and a stent with a membrane. Through endovascular aneurysm exclusion, the aneurysm is excluded from the parent artery, and then the aneurysm is occluded to realize reconstruction of the parent vessel. This stent provides a new and efficient treatment method for traumatic pseudoaneurysm in the intracranial segment of the internal carotid artery. From December 2013 to May 2019, 15 patients with traumatic pseudoaneurysm of the intracranial segment of the internal carotid artery were treated with intracranial WCS in the Department of Neurosurgery, General Hospital of PLA Central Theater Command, with good results. This treatment is retrospectively reported in the current study.

## Methods

The ethics committee of our institution granted ethics approval of this study and waived the requirement for written informed consent. In total, 15 consecutive confirmed carotid pseudoaneurysm patients who were treated with WCS at our department between December 2013 and May 2019 were enrolled. The patients included in this study met the following criteria:

Clinical data: Of the 15 patients, 13 were male and 2 were female; the mean age was 39.0 ± 10.7 years (range, 15–51 years). Eleven patients had a clear history of trauma: 6 cases had car accident injury, 3 cases had fall-from-height injury, and 2 cases had heavy object injury; 2 cases involved internal carotid artery injury during brain tumor surgery; 1 case had a history of head and neck radiotherapy for nasopharyngeal carcinoma; and 1 case involved residual pseudoaneurysm after balloon treatment of traumatic carotid cavernous fistula. The clinical manifestations of the 15 patients were recurrent massive nasal bleeding in five patients, recurrent intracranial hemorrhage in six patients, and stroke in two patients. The neurological function status of each patient at the time of admission and follow-up was assessed and graded as good for Modified Rankin Scale (MRS) scores of 0 to 1 and poor for MRS scores of 2 to 5 and death. All patients or their families gave written informed consent.

**Figure 1:**
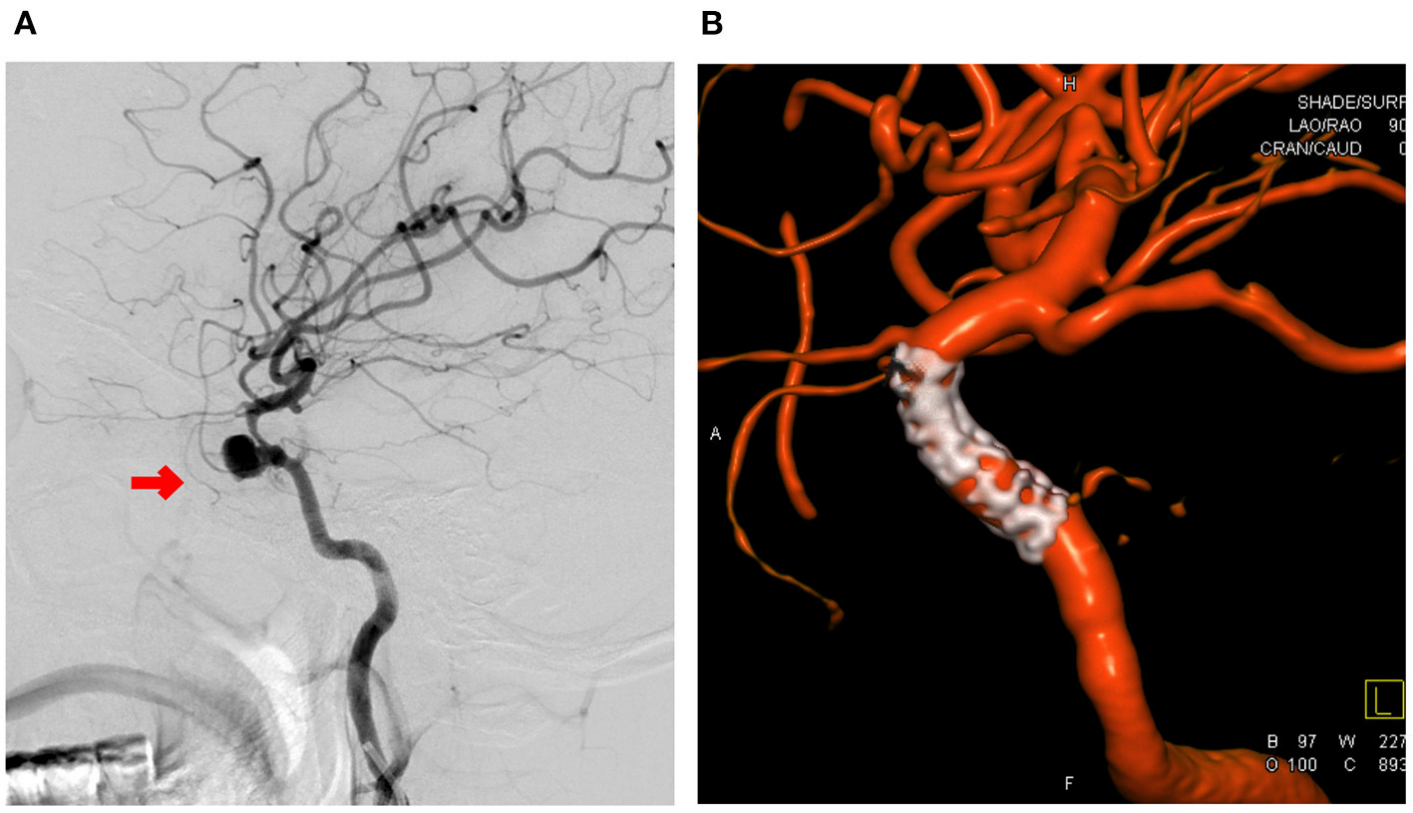
A 38-year-old female patient was admitted to the hospital 2 weeks after resection of sphenoid ridge meningioma. **(A)** Preoperative digital subtraction angiography (DSA) examination showed pseudoaneurysm in the cavernous segment of the internal carotid artery. The straight diameter of the blood vessel at the distal and proximal ends of the parent artery was 2.1 and 2.9 mm, and the size of the tumor was 8.4 × 7.8 mm. Intraoperative placement of a Willis covered stent followed by DSA examination showed resolution of the pseudoaneurysm. **(B)** Shows that the stent graft had good compatibility and patent vessels.

**Figure 2:**
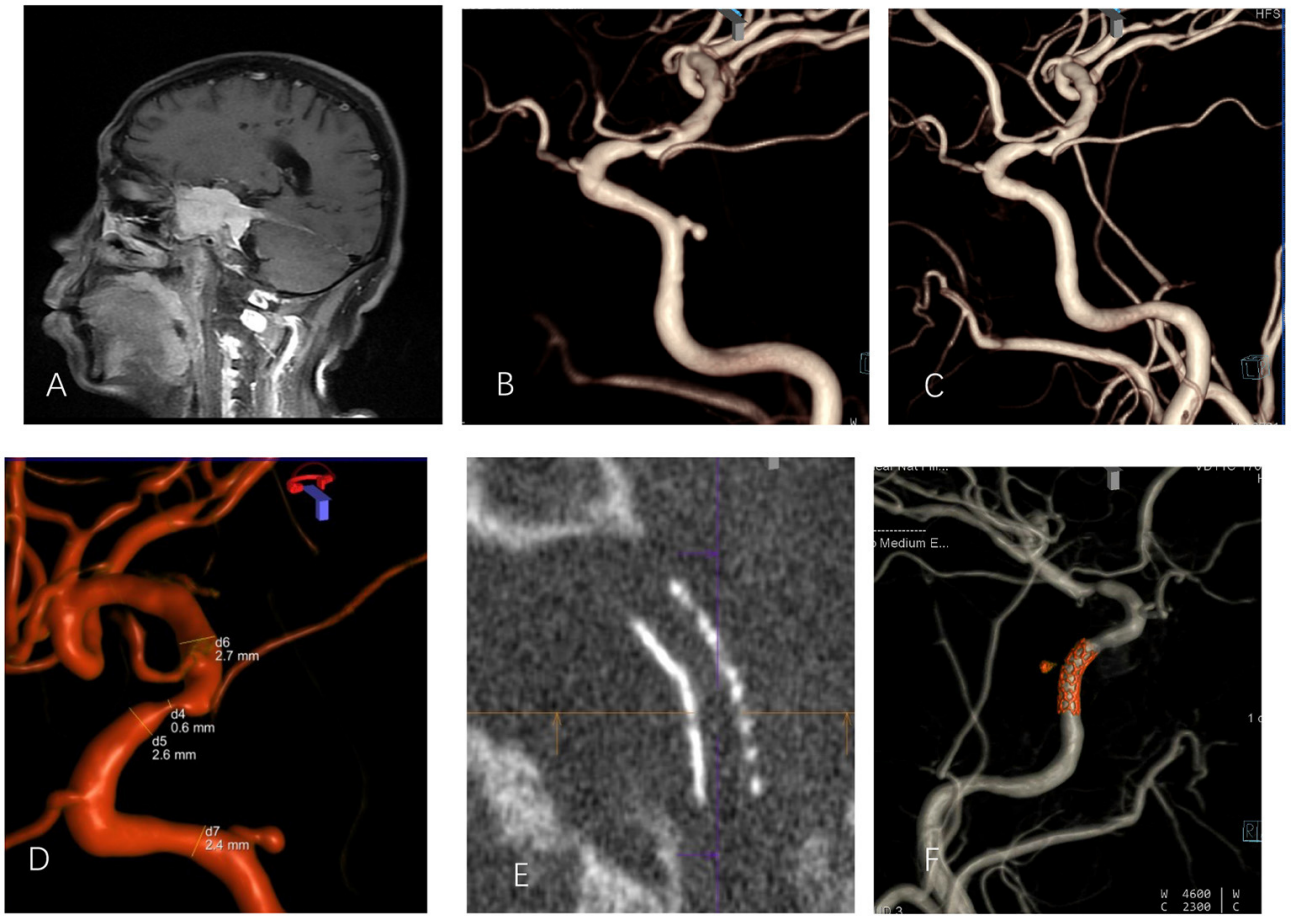
A 51-year-old male with intraoperative internal carotid artery injury of right sphenoid clival meningioma and postoperative secondary right internal carotid cavernous pseudoaneurysm before and after interventional therapy **(A)**. Enhanced MRI revealed that the tumor wrapped the right internal carotid artery. **(B)** A DSA-3D lateral image showed pseudoaneurysm of the posterior wall of the cavernous sinus of the right internal carotid artery. **(C)** The pseudoaneurysm disappeared after stent treatment. **(D)** Measurement of the diameter of the parent artery; **(E)** Maximum intensity projection showed that the stent opened completely. **(F)** Postoperative dual-volume imaging showed that the stent graft had good compatibility and that the vessel was patent.

**Figure 3:**
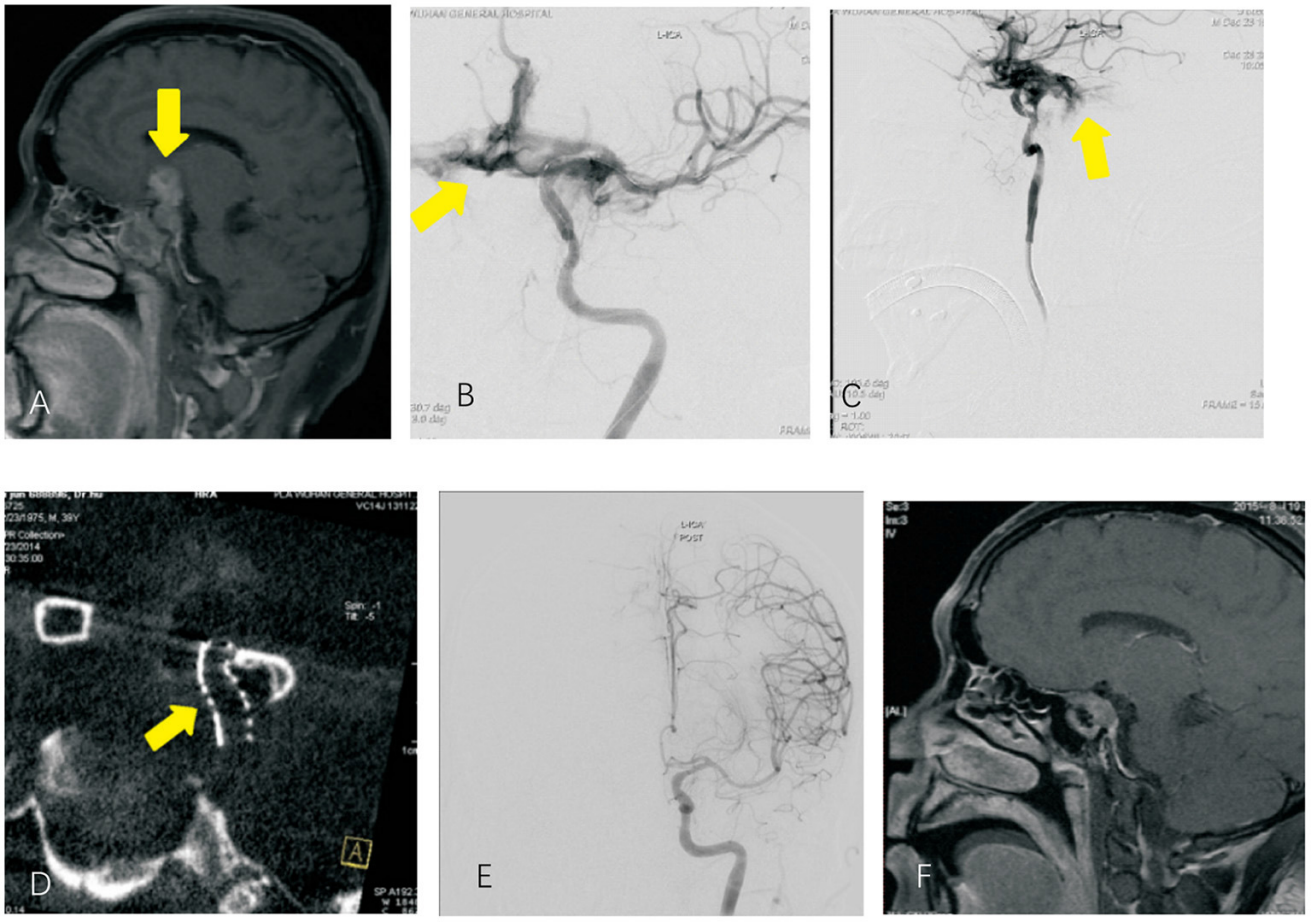
Invasive pituitary adenoma and intraoperative internal carotid artery rupture **(A)**. Anterior MRI sagittal image, which showed pituitary adenoma; **(B,C)** DSA image of the ophthalmic segment of internal carotid artery rupture, which showed contrast agent spillage from the rupture to the arachnoid membrane; **(D)** maximum intensity projection showed good apposition of the Willis covered stent; **(E)** successful repair of the internal carotid artery after stent implantation; **(F)** postoperative MRI suggesting tumor resection.

Imaging data (Figures 1–3): All patients were diagnosed with traumatic pseudoaneurysm of the intracranial segment of the internal carotid artery by preoperative digital subtraction angiography (DSA). The results of three-dimensional reconstruction showed that the maximum diameter of 15 pseudoaneurysms in 15 patients was 7. 5 ± 2.2 (1.7 to 16.0 mm), neck width was 3.5 ± 1.9 (0.5 to 7.7) mm, and the distal and superior vessel diameters of the parent artery were 2.6 ± 0.8 (1.1 to 3.6 mm) and 3.0 ± 0.7 (1.2 to 4.0 mm), respectively. Of the 15 pseudoaneurysms, 4 were close to the ophthalmic artery, 5 were close to the posterior communicating artery, and the remaining 6 were located in the cavernous sinus segment without important vessel proximity.

### Treatment and Follow-Up

Surgical methods (Figures 1–3): All patients received oral antiplatelet aggregation drugs (Bayaspirin 100 mg/d, clopidogrel 75 mg/d) 3 days before surgery. During the operation, the patient was placed in the supine position with local anesthesia at the puncture site, the femoral artery was punctured by the Seldinger method, and an 8F sheath was placed. After completion of whole cerebral angiography, the patient was transitioned to general anesthesia, with systemic heparinization. Aided by a 0.035-inch guidewire, the coaxial catheter system consisting of an Envoy guiding catheter and Navien catheter was delivered to the internal carotid artery as close to the lesion site as possible. The Envoy 8F guiding catheter was placed at the C2 level of the internal carotid artery. Under the guidance of a roadmap, the Navien catheter was navigated to the cavernous segment of the internal carotid artery or even higher via an Echelon 10 microcatheter and microwire. DSA was performed on the lesion side, and three-dimensional reconstruction was performed again to measure the distal and superior diameter of the patent artery, the size of the aneurysm and the width of the neck. A WCS of appropriate size was selected based on the diameter of the parent artery and the length of the involved diseased vessel. None of the 15 patients included in this study had internal carotid artery occlusion. Under the guidance of a microwire, the stent was quickly moved to the end of the Navien catheter, and its position was adjusted by projection at multiple angles to make sure that the stent covered the aneurysm neck and avoided the opening of the branch artery. After finalizing the position, the balloon was slowly filled, the filling pressure was maintained at the maximum diameter of the stent for ~10 s to keep the stent completely expanded, and then the pressure was released.

DSA was performed again after emptying the balloon. If the aneurysm completely disappeared immediately, the balloon was withdrawn; DSA was performed after 10 min of observation to ensure that the parent artery was patent. If there was “endoleak,” the balloon could be inflated again until the endoleak disappeared. If contrast medium continued to enter the aneurysm, follow-up observation or postoperative auxiliary compression of the carotid artery on the lesion side was performed for 30 min, according to the endoleak findings, once or twice a day for 1 to 2 months. A total of 15 WCS (Shanghai Shentong Technology Co., Ltd., China) were placed in 15 patients as follows: 3.5 mm × 7.0 cm (*n* = 2), 3.5 mm × 10.0 cm (*n* = 3), 3.5 mm × 13.0 cm (*n* = 2), 3.5 mm × 16.0 cm (*n* = 1), 4.0 mm × 7.0 cm (*m* = 1), 4.0 mm × 13.0 cm (*n* = 5), and 4.5 mm × 7.0 cm (*n* = 1). After the operation, all patients underwent routine cranial Dyna CT reexamination and stent reconstruction.

#### Postoperative Treatment

After surgery, all patients were continuously infused with 2 to 4 ml of tirofiban using a micropump. Beginning the next day, treatment with oral clopidogrel 75 mg/day and Bayaspirin 100 mg/day was administered for 3 months. After 3 months, treatment was changed to oral Bayaspirin 100 mg/day only for 6 months. One week after surgery, platelet activation function and thromboelastography were repeated.

#### Follow-Up Methods

All patients were followed up in the outpatient department after discharge, including reexamination by DSA to determine whether the aneurysm recurred and whether there was stenosis of the parent artery.

### Statistical Analyses

A Pearson chi-square test was applied to compare the categorical variables between the groups. All analyses were performed with SPSS software, version 24.0 (International Business Machines Corp., Almond, NY, USA), and *p* < 0.05 was considered statistically significant.

## Results

### Immediate Postprocedural Results

Of the 15 patients, two patients were scored as neurological function good, and 12 patients were scored as neurological function poor on admission. Twelve were successfully implanted with a WCS in one procedure; the other 3 patients had endoleak after implantation of a WCS, and of these patients, 1 underwent balloon dilatation once, which resolved the endoleak, and 2 underwent auxiliary compression on the ipsilateral common carotid artery after surgery. Immediate postoperative DSA showed successful vascular reconstruction and resolution of the pseudoaneurysm in all patients (Figures 1–3). One patient died of severe traumatic brain injury after the operation, and the other 14 patients underwent postoperative reexamination of cranial Dyna CT. No new cerebral infarction or cerebral hemorrhage was observed. None of the patients had surgery-related complications.

### Follow-Up Results

Clinical follow-up data and DSA were collected for 14 of the patients; the mean follow-up period was 5.8 ± 1.2 months. None of the 14 patients had any recurrence of aneurysms or stenosis of the parent artery throughout the follow-up period. No ischemic or hemorrhagic event was reported by any patient during the follow-up period. The functional neurological status was good (mRS score 0 or 1) in 13 patients, and poor (mRS score 2–5) in 1 patient at follow up. And the neurological function status of each patient at the time of follow-up were better than admission according the mRS score (*p* < 0.001).

## Discussion

Traumatic pseudoaneurysm of the intracranial segment of the internal carotid artery usually occurs after blunt or acute trauma to the head and neck. Vascular wall injury or arterial rupture bleeding often appear, especially when pseudoaneurysm is combined with anterior cranial fossa fracture, and blood flows from the rupture to form a hematoma. The rupture is gradually closed by a blood clot, which promotes temporary hemostasis. Then, the hematoma dissolves. At the same time, the surrounding tissue gradually organizes to form a fibrous tissue capsule, ultimately leading to the formation of a pseudoaneurysm. Over time, under the impact of the continuous pulsatile blood flow of the parent artery, the pseudoaneurysm capsule wall expands, increasing in size, and eventually ruptures and bleeds again.

Once a traumatic pseudoaneurysm of the internal carotid artery is diagnosed, it should be treated as early as possible before it ruptures. The treatment principle is to repair the damaged vascular wall, isolate the blood circulation of the aneurysm, keep the parent artery unobstructed, and retain the important branches of the parent artery. Traumatic pseudoaneurysm in the intracranial segment of the internal carotid artery is usually located deep in the head, close to the skull base, and the trauma will lead to disorder of the normal anatomical structure of the ICA; the traditional surgical treatment is difficult, the risk is high, and prognosis is poor. In recent years, interventional therapy has been used to treat traumatic internal carotid artery pseudoaneurysm because of its advantages of minimal trauma and a good curative effect (2).

A covered stent can directly block the aneurysm neck with the biophysical membrane on its surface and then isolate the aneurysm from the blood circulation. At the same time, through reconstruction of the lumen of the parent artery, the pressure in the aneurysm cavity can be reduced, the original hemodynamics can be restored, and a blood clot can form in the aneurysm cavity over time until occlusion (3). Covered stents are the most ideal therapeutic material for pseudoaneurysm of the ICA. A total of 15 patients with traumatic pseudoaneurysms in the intracranial segment of the internal carotid artery were included in this study. According to the diameter of the parent artery and the length of the involved vessels, an appropriately sized WCS was selected for embolization. The immediate occlusion rate of pseudoaneurysm was higher in this study than in previous reports. At the same time, the incidence of intracranial rebleeding and postoperative ischemic events caused by surgery was low, and the long-term recurrence rate was low.

In the literature, many complications, such as long-term stenosis of the vascular lumen, stent collapse into the pseudoaneurysm lumen during operation and long-term endoleak, have been reported after using WCSs (3–5). According to these reports, the potential causes of endoleak include mismatch between the model of the WCS and diameter of the parent artery, uneven lumen of the diseased vessel, incomplete coverage of the aneurysm neck, transient vasospasm, stent displacement and stent rupture (6, 7). In this study, the incidence of endoleak in the treatment of intracranial traumatic pseudoaneurysm of the internal carotid artery with a WCS was 3/15. One patient was treated with balloon dilatation, and the other 2 patients were treated with carotid artery compression. The leakage disappeared after 4 months of follow-up. In our experience, when angiography immediately after the operation indicates trace contrast agent entering the aneurysm cavity, we can compress the carotid artery in some patients. A long-term endoleak can disappear by itself, but there is also the risk of another rupture. Therefore, it is suggested that patients under long-term observation should be followed up regularly by cerebral angiography until the endoleak disappears.

The closure of side branches or perforating arteries originating from the covered segment of the artery has always been a major concern in the use of covered stents for cerebral aneurysm treatment. Some reports suggest that the ophthalmic artery (OA) can be sacrificed if necessary because reconstruction of the OA from external carotid artery collaterals is possible (8, 9). The anterior choroidal artery (AchoA) is another important artery that primarily feeds the area of the optic tract, internal capsule, and cerebral peduncle. In our study, before stent deployment in the C7 segment, we carefully evaluated the angiogram from multiple angles to prevent covering the ostium of the AchoA. There were no complications of occlusion of branches or perforating vessels in 15 patients. This may be due to appropriate case selection and accurate stent release.

Late in-stent stenosis during long-term follow-up is another complication of covered stents (10). In-stent stenosis might be caused by neointimal tissue proliferation, and there was no difference in the postintervention or follow-up lumen (at the junction of the two stents) when overlapping stents were compared with non-overlapping stents (11). Standardized antiplatelet aggregation therapy has been proven to play an important role in anti-intimal hyperplasia (12). Moreover, poor compliance with antiplatelet aggregation drugs has been reported to be an independent risk factor for stent stenosis. In this study, 12 patients were followed up by DSA. Through the last follow-up, none of the patients had any recurrence of aneurysms or stenosis of the parent artery. This finding is mainly related to strict treatment with antiplatelet aggregation drugs.

In conclusion, due to the lack of a normal arterial wall, traditional interventional treatment cannot effectively repair the rupture, and even at the cost of occlusion of the parent artery, it is not the best treatment. A covered stent has the advantages of immediate reconstruction of the normal vascular wall, preservation of the patency of the parent artery and restoration of normal blood perfusion, indicating that it is an ideal treatment method. Longer-term follow-up and additional clinical experience are needed to fully determine the safety and efficacy of this device.

## Limitations

Our study has several limitations. First, all patients were enrolled from a single center, and a potential selection bias regarding region and race may have occurred. Our study is a retrospective study, and larger samples and longer follow-up studies will be needed to validate our findings.

## Data Availability Statement

The raw data supporting the conclusions of this article will be made available by the authors, without undue reservation.

## Ethics Statement

Written informed consent was obtained from the individual(s) for the publication of any potentially identifiable images or data included in this article.

## Author Contributions

All authors listed have made a substantial, direct and intellectual contribution to the work, and approved it for publication.

## Funding

This study was supported by Grants from the Hubei Provincial Natural Science Foundation of China (Grant No. 2020CFB125).

## Conflict of Interest

The authors declare that the research was conducted in the absence of any commercial or financial relationships that could be construed as a potential conflict of interest.

## Publisher's Note

All claims expressed in this article are solely those of the authors and do not necessarily represent those of their affiliated organizations, or those of the publisher, the editors and the reviewers. Any product that may be evaluated in this article, or claim that may be made by its manufacturer, is not guaranteed or endorsed by the publisher.
